# Experiences of GPs with incretin mimetics: a qualitative study in Germany

**DOI:** 10.3399/BJGPO.2025.0135

**Published:** 2026-04-22

**Authors:** Amelie Ege, Nicole Lindner, Nele Kornder, Annika Viniol, Veronika van der Wardt

**Affiliations:** 1 Department of General Practice/Family Medicine, Marburg University, Marburg, Germany

**Keywords:** obesity, qualitative research, type 2 diabetes, primary healthcare

## Abstract

**Background:**

Incretin mimetics are increasingly used to manage diabetes and to support weight loss in people with obesity. However, their application by GPs in primary care settings remains underexplored.

**Aim:**

This study aimed to investigate GPs’ healthcare experiences in relation to incretin mimetics, such as semaglutide.

**Design & setting:**

A qualitative study using semi-structured interviews with GPs from Hesse in Germany, recruited through purposive sampling. Data were collected between July 2024 and May 2025.

**Method:**

We conducted 15 semi-structured interviews. Data were analysed using Braun and Clarke’s thematic analysis approach.

**Results:**

We identified six key themes: incretin mimetics as a new treatment option in the treatment of diabetes and obesity; patient expectations; the media as a key driver; challenges related to financial accessibility and supply; patients’ therapy adherence; and the effects on quality of life and lifestyle changes.

**Conclusion:**

Our findings emphasise that facilitating patient education using supportive educational materials might counter the lack of reliable information. Moreover, collecting both qualitative and quantitative data on incretin mimetic usage from the patient perspective would be valuable for a more comprehensive understanding of the use of the medication.

## How this fits in

Incretin mimetics are increasingly used for type 2 diabetes and obesity, but qualitative evidence from German primary care is scarce. This study provides new insights into GPs’ perspectives, showing high, media-driven patient demand; perceived effectiveness and adherence; and challenges around patient self-payment, supply shortages, and prioritisation between indications. The findings highlight the need for reliable patient information, and further research to support safe and equitable use.

## Introduction

Diabetes and obesity are major global health challenges. The International Diabetes Federation estimates that 11.1% of adults currently live with diabetes, rising to one in eight by 2050, representing a 46% increase.^1^ Recent analyses projecting current trends into the future estimate that within the next 25 years, one in three young people worldwide could be overweight or obese.^2^

These trends highlight the need for effective therapy and management. Incretin mimetics have shown great promise in clinical studies: a meta-analysis published by Li *et al* in 2024 showed that semaglutide plus metformin significantly improved glycaemic markers, body mass index (BMI), insulin resistance, and lipid profiles in people who are overweight and have type 2 diabetes.^3^ New incretin mimetics are currently in development and have demonstrated promising improvements in HbA1c levels.^4^ Clinical trials show a lowered cardiovascular risk^5^ and potentially reduced renal and cardiovascular events in patients with diabetes who have chronic kidney disease.^6^

Furthermore, their use has led to weight loss of up to 25% over 70–90 weeks.^7^ In comparison, standard diets can achieve 10% weight loss in 6 months, but this is often followed by weight regain.^8^ In a study published in 2021, semaglutide reduced body weight by 14.9% and improved metabolic health in patients with obesity.^9^ However, discontinuation is commonly followed by weight regain, underlining the need for sustained treatment or lifestyle strategies.^10^ Gastrointestinal side effects such as nausea, vomiting, and diarrhoea are common; rarer risks include gallbladder disorders and acute pancreatitis.^11^ While quantitative evidence on incretin mimetics is well established, qualitative data from primary care remain limited.

A Swedish qualitative interview study (student thesis, not peer-reviewed) among patients with type 2 diabetes and healthcare professionals found high satisfaction with glucagon-like peptide-1 (GLP-1) agonists owing to stable blood sugar levels and the convenience of weekly injections.^12^ While most patients felt they had a sufficient understanding of their treatment, regular follow-ups and clearer explanations could further enhance motivation and reduce anxiety.^12^

A Danish qualitative study by Andreassen *et al* ^13^ interviewed GPs and nurses about their experiences with semaglutide. The study revealed strong trust in the drug’s efficacy. Social and financial barriers influenced access to treatment, with wealthier patients being more likely to afford the therapy. Patients were often proactive, sometimes complicating shared decision making and resulting in prescriptions frequently being given despite concerns.^13^

In Germany incretin mimetics have been prescribed for type 2 diabetes since 2006, as add-ons to metformin when glycaemic targets cannot be achieved with monotherapy, or as initial combination in patients with clinically relevant cardiovascular disease.^14^ In this indication, treatment is reimbursed by health insurance providers.^15^ For obesity (BMI ≥30 or ≥27 with comorbidities), semaglutide has been approved since 2022 but reimbursement is currently not provided.^15,16^ The prescribing process is, apart from cost coverage, comparable across both indications: prescriptions are often issued by GPs and diabetologists, though initiation by physicians from other specialties is also possible.

Media coverage has driven rising demand for GLP-1 agonists,^17^ and research is widely discussed beyond scientific journals.^18^ A study using Google Trends found exponential growth in searches for ‘Ozempic’ in 2023.^19^ Social media plays a key role, with viral content promoting the drugs while rarely mentioning side effects.^20^ Often referred to as the ‘weight loss injection’, off-label use for weight reduction increased before Ozempic’s approval, resulting in supply shortages.^19^

Despite their key role, little is known about GPs’ experiences with incretin mimetics in Germany.

The aim of this study was to explore the experiences and views of GPs in Germany regarding the use of incretin mimetics in everyday primary care practice.

## Method

### Design, setting, and participants

This qualitative, semi-structured interview study included GPs practicing in eastern, central, and northern Hesse, Germany. Physicians in training, retired doctors, and those not currently working in general practice settings were excluded.

### Sampling and recruitment

A purposive sampling approach was used to ensure a diverse selection of GPs based on practice size, location, specialisation, and experience. Practices were identified through the research network of primary care practices affiliated with the Department of Primary Care, Marburg University and practice lists of the German Association of Statutory Health Insurance Physicians in the state of Hesse (otherwise known as KV Hessen). Interested GPs contacted the study team directly, and participation was voluntary.

Specialisations in German primary care include fields such as nutritional medicine, diabetology, or palliative care. We included GPs from both diabetology-focused and general practices, with and without a personal specialisation in diabetology to reduce bias from participant characteristics. Participants did not review their transcripts or provide feedback on the analysis to reduce participant burden (no member checking).

### Sample size and data saturation

Sample size was determined with reference to both data saturation and the concept of information power. Though saturation may not fully align with reflexive thematic analysis,^21^ we used it pragmatically to judge when no substantially new insights emerged. Following Malterud *et al*, adequacy was further determined by the research aim, data richness, and analytic strategy, with data collection ending once additional interviews yielded only minor variations.^22^

### Study procedures

Data collection was conducted between July 2024 and May 2025.

After recruitment via email and phone, interested GPs received study information, a short questionnaire, and a consent form via email or by post with a pre-paid return envelope. Signed informed consent was obtained before the interviews started.

### Researcher characteristics and reflexivity

The interviews were conducted via telephone. The interviews were carried out by the first author (AE), a fifth-year medical student at Marburg University, who conducted the study as part of her doctoral thesis. The first author had no prior relationship with the participants. AE was supported by the second and third authors (NL and NK, both academic GPs). The fifth author (VvdW), who has a background in psychology and extensive experience in qualitative research, supervised the interviewer.

### Interview guide

A structured interview guide (overview in Table 1; full version in Supplementary material) ensured a consistent yet flexible discussion and was developed by the interviewer in collaboration with the fourth fifth (VvdW). The semi-structured format allowed question order adaptation while covering key topics, such as patient adherence. Some questions specifically addressed diabetes or obesity, while others were kept open to allow participants to elaborate more broadly.

**Table 1.** Interview guide: overview of interview topics and sample questions

Topic	Example questions from the interview
**Incretin mimetics in daily practice**	Have you ever been explicitly asked about semaglutide? To what extent does media coverage influence your handling of these medications? How well informed do you feel about semaglutide and its use?
**Use in diabetes treatment**	What experiences have you had? How do you assess therapy adherence?
**Use in obesity treatment**	To what extent do the costs of the therapy, which are borne by the patients, influence its use? How do you evaluate pharmacological treatment of obesity with semaglutide as an alternative to surgery?

The interview guide was refined through pilot interviews with three GPs, which served to clarify, structure, and consolidate the interview questions, but were not included in data collection. Interview questions were discussed in the departmental qualitative research working group, which includes practicing GPs. Additionally, three of the co-authors (NL, NK, AV), who are primary care clinical academics, reviewed the interview schedule before interviews commenced.

### Data collection

The interviews were recorded and transcribed according to the adapted transcription rules by Dresing and Pehl.^23^ A research diary captured the interviewer’s impressions.

A short questionnaire collected data on GP practices and participants to provide context for the responders’ experiences. It included practice size, approximate number of cases per year, number of physicians in the practice, practice location (rural or urban), years of professional experience as a GP and specialisations.

### Data analysis

The questionnaire data were analysed descriptively to characterise the sample.

The analysis followed the thematic analysis approach by Braun and Clarke.^24^ The research team each read their assigned transcripts multiple times to familiarise themselves with the data, and relevant GP experiences were highlighted and noted. Notes from the interviews were also reviewed, reflecting the interviewer’s subjective impressions of significant aspects.

MAXQDA (version 24.4.0) was used for transcription, structuring, and coding. Audio files were transcribed with partial support from the integrated AI-based transcription feature and checked by the interviewer.

Segments were manually coded, refined through subcodes, and developed iteratively into broader themes. Codes were based on relevant data features,^25^ visualised with colours, thematic maps, flow charts, and adjusted over time; similar quotes were compared to form preliminary themes. Themes were formed by grouping related codes, which were broader and less specific than individual codes.^26^

Finally, the analysis was reviewed for alignment with the data and research question. The process combined inductive and deductive approaches, with interview guide adjustments made during data collection. The research findings were documented in the final report.

The coding framework was developed by the first author (AE) in consultation with the fifth author (VvdW), and coding decisions were regularly discussed within the study team of all authors.

Preliminary codes, themes, and the study design were presented to the department’s qualitative group and a patient participation group of local citizens with primary care (those with regular and frequent attendance) and chronic-illness experience. This aimed to ensure patient-oriented perspectives and clarity in research. Their feedback informed data collection and analysis, though, to our knowledge, none had personal experience with incretin mimetics. The study’s co-authors helped to refine and validate themes and strengthen analytical depth.

Credibility, dependability, confirmability, transferability, and reflexivity^27^ were evaluated to examine result trustworthiness.

All quotes were translated into English for publication and reviewed by the fifth author (VvdW), who is bilingual in German and English.

### Trustworthiness

The study ensured trustworthiness through methodological transparency, peer debriefing, purposive sampling, patient involvement, and critical self-reflection. Purposive sampling and contextual detail supported transferability, though the study was limited in sample size. Broader inclusion of regions and specialties could enhance this further. Systematic documentation of the research process, including the interview guide (see supplementary material), transcription rules, and analysis criteria, ensured the study’s reproducibility.

To support credibility and reduce bias, results were discussed with the department’s qualitative research group and the patient participation group. Presenting preliminary findings helped sharpen the analysis and add contextual understanding. Confirmability was supported by direct quotes and ongoing self-reflection to reduce bias.

## Results

The sample consisted of 15 GPs from central, eastern, and northern Hesse. Participants had between 6 and 28 years of experience, treated 6000 to 14 000 cases annually, and worked in rural and urban areas. Some GPs had additional qualifications in fields such as diabetology, internal medicine, or naturopathy (see Table 2).

**Table 2.** Participant characteristics

ID	Physicians practice, *n*	Annual case load, *n*	Practice setting	Experience, years	**Specialisations**
T01	2	10 000	Rural+urban	17	Endocrinology, diabetology
T02	6 (3+3)^a^	14 000	Rural	6	None
T03	3 (1+2)	10 000	Rural	28	Occupational medicine
T04	1	7750	Rural	28	Sports medicine, manual therapy, naturopathy
T05	4 (1+3)	6000	Rural	23	Naturopathy, acupuncture
T06	6 (2+4)	10 000	Rural	8	None
T07	5 (3+2)	14 000	Urban	16.5	Diabetology
T08	6 (4+2)	14 000	Urban	8	None
T09	1	8000	Rural	11	None
T10	4 (2+2)	8000	Urban	13	None
T11	4 (2+2)	9000	Rural	6	None
T12	4 (2+2)	9000	Rural	6	None
T13	5 (3+2)	12 400	Rural	18	Naturopathy
T14	6 (4+2)	16 000	Rural	15	Naturopathy, chiropractic, occupational medicine
T15	5 (2+3)	11 200	Rural	9	None

a Number of owners + number of employees.

### Thematic structure

Interviews revealed six key themes:

1. incretin mimetics as a new treatment option in the treatment of diabetes and obesity;
2. patient expectations;
3. the media’s influence as their key driver;
4. challenges related to financial accessibility and supply shortages;
5. patients’ therapy adherence; and
6. the effects on quality of life and lifestyle changes.

Figure 1 shows themes and the relationships between them.

**Figure 1.**
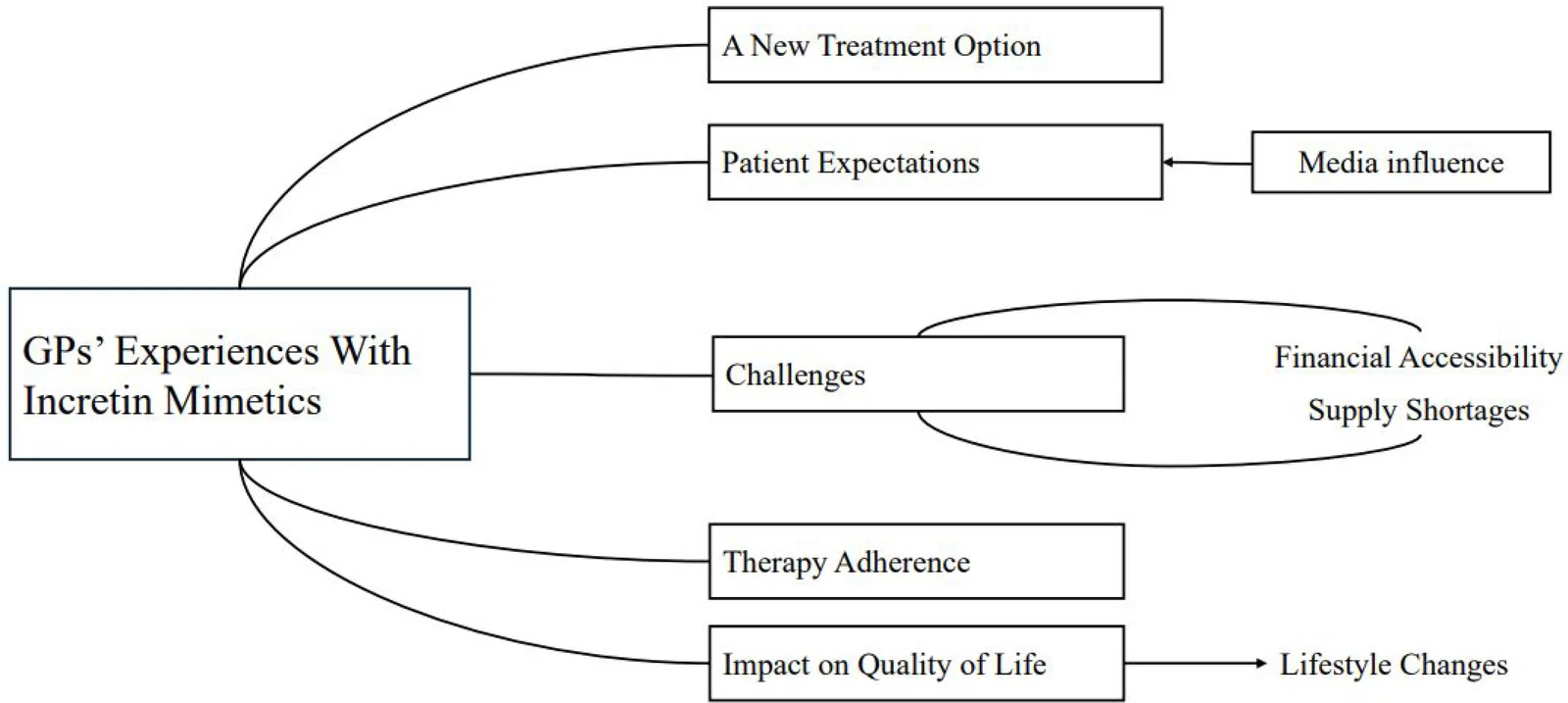
Themes, subthemes and their relationship

### A new treatment option

The interviews indicated that treating diabetes and obesity was often perceived as complex and unsatisfactory. Particularly in diabetes care, side effects, and dissatisfaction with existing therapies were highlighted. One GP described insulin treatments as ‘*blood sugar cosmetics’* (T01)*,* indicating that they provided little long-term health improvement.

Incretin mimetics expanded therapeutic options and were seen not merely as an addition but as a fundamentally new approach to what had often been frustrating treatment. While incretin mimetics are not entirely new, their growing relevance, especially through their role in obesity treatment, was reflected in the interviews:

*‘And these incretin hormones have represented a real turning point for these patients.’* (T01)

GPs considered the medication a relief for patients, with ease of use being a key advantage. In this context, they were often compared to insulin, highlighting their significance especially in difficult-to-manage diabetes:

‘*It’s better than having to inject insulin multiple times a day and measure blood sugar.*’ (T08)

*‘When I started, it was insulin, insulin, insulin and nothing else. People just kept gaining weight. Now, things are completely different.’* (T07)

While most accounts emphasised the benefits, some GPs mentioned concerns about potential side effects, especially in the early phase of use.

Though incretin mimetics can be prescribed for obesity, they are not covered by German health insurance. Many patients still regard them as the preferred option:

*‘For many* [patients]*, this is often the first therapy option.’* (T10)

This is particularly relevant given the challenges in obesity management, where GPs often feel limited in supporting patients. They emphasised the burden experienced by patients with obesity, framing it as a disease rather than individual responsibility. In Germany, bariatric surgery remains the last resort for treating obesity and is reimbursed under certain indications. Opinions among GPs varied, but some viewed incretin mimetics as a viable alternative:

*‘I mean, I also see the successes with surgeries.’* (T02)

*‘… people on GLP-1 analogues sometimes actually achieve weight loss comparable to that of bariatric surgery.’* (T07)

*‘This can indeed be an alternative because it is not a one-way street* … *Those kinds of procedures are surgical interventions with significantly more risks than Ozempic.’* (T04)

### Patient expectations

GPs reported a shift in traditional roles, with patients actively requesting incretin mimetics, particularly driven by demand for weight reduction:

‘... *these were actually all active requests from the patients.*’ (T13)

According to GPs, different patient groups actively inquired, such as patients with diabetes:

*‘The desire for weight reduction and HbA1c lowering is expressed, with the patient actively asking.’* (T02)

Even patients with well-controlled diabetes expressed interest in the treatment for weight loss. However, GPs emphasised that patients with obesity were more likely to actively inquire:

*‘It’s always patients with obesity who heard about it and then ask.’* (T11)

Some patients without medical indication sought the drug for aesthetic reasons:

*‘I had a few who weren’t obese or diabetic, just unhappy with a bit of belly fat, wanting to lose it quickly.’* (T06)

In these cases, GPs stressed that such use lacked a valid medical indication and evaluated these requests critically, emphasising the need for comprehensive patient education on therapeutic indications, risks, and limitations. While some GPs had the impression that patients perceived this as a ‘*lifestyle product*’ (T03)*,* others pointed out: *‘the people who wanted to do it for lifestyle reasons were rather rare’.* (T04)

Given the unusually high demand, GPs observed that patients often knew incretin mimetics by name, in contrast to most other medications (with which patients are usually less familiar and engage less actively with). Yet patients still had limited understanding of incretin mimetics’ risks, limitations, or payment aspects:

*‘They know nothing about the mechanisms, they might not know about the side effects either; this is the only signal they have: there’s something where I can lose weight. I want that.’* (T01)

*‘… you have to keep using it if you want the effect to last. They often don’t know that.’* (T06)

*‘When you explain to them that the health insurance doesn’t cover it for the indication of obesity, it can get tricky.’* (T07)

GPs saw both informed and uninformed patients:

*‘There are many very informed patients, but there are also a lot of naive ones who just heard somewhere that this treatment exists and that the doctor can simply prescribe it.’* (T08)

Overall, GPs encountered high expectations: patients with obesity hoped for easy prescriptions and quick weight loss, while those with diabetes expected straightforward improvement of disease control. GPs reported feeling forced to disappoint these patients by explaining side effects, supply shortages, and the need for self-payment in the treatment of obesity, or specific prescription criteria in the treatment of diabetes (see Introduction):

*‘And this is definitely part of the education, for which I take some time to highlight the downsides of this medication …’* (T04)

### Media as the key driver

The medication’s media presence was perceived by GPs as ‘*above average*’, driving patient demand particularly in the context of obesity treatment and for aesthetic reasons. Some GPs perceived the media portrayal as an invitation to patients to ask for the medication:

*‘Just go to your doctor, get it. And it’s all so simple, and it works.’* (T01)

The media presence was viewed critically:

*‘… because this medication is being portrayed completely incorrectly … in the press, it’s presented as if this is the weight-loss injection, available for everyone.’* (T01)

GPs had the impression that patients identified and compared themselves to celebrities, hoping not only for weight reduction but also for an associated overall lifestyle change:

*‘… this is the medication for the rich and the beautiful. And I want that too.’* (T07)

It was emphasised that patients’ knowledge was often shaped more by wishful thinking and amplified by social media than informed by facts. GPs furthermore noted:

*‘I do believe that patients who suffer from obesity and are trying to do something about it are also naturally looking for solutions, are more susceptible, and are probably then targeted by these algorithms.’* (T05)

### Challenges

#### Financial accessibility

Considering that incretin mimetics for obesity must be paid for privately, GPs reported that patients’ ability to access treatment under this indication varied depending on their financial situation. Many patients initially showed interest but wealthier patients were more likely to opt for the treatment, while those in disadvantaged regions often couldn’t afford it:

*‘In the practice I had in* [rural area]*, for example, most were older people, … not working and immigrants, et cetera, who were not financially well off. Many of them could not afford it. In our practice in* [wealthy urban district]*, we had more private clientele. There were more patients who could afford such treatments.’* (T01)

*‘And that did deter some, or the majority, especially those with lower incomes.’* (T02)

Long-term success – understood by GPs as sustained weight loss through ongoing use of the medication for obesity treatment – was therefore reported to be more common among patients with financial means. GPs critically questioned why obesity-related complications were reimbursed, but not obesity treatment itself:

*‘It would actually be logical to approve it for obesity treatment at the expense of statutory health insurance.’* (T07)

They noted that pharmacological weight loss treatments require long-term use to prevent regain, yet argued that this should be seen in parallel with obesity-related comorbidities, which themselves necessitate life-long treatment.

Some GPs expressed concern that patients who were prescribed incretin mimetics as part of their diabetes therapy (covered by health insurance) might resell them:

*‘And I have to say, the fear that it might be resold, that’s exactly the point.’* (T12)

Though lacking evidence, this concern remained present.

GPs noted that health insurers in Germany closely monitored prescriptions as a form of quality control, as incretin mimetics are reimbursed and high-cost compared to other diabetes medications, and therefore checked for approved indications and cost-control compliance:

*‘I think the insurance companies are watching very closely.’* (T07)

#### Supply shortages

Another challenge reported by GPs was supply shortages, which particularly affected patients with diabetes who were dependent on incretin mimetics:

*‘The only thing that really annoys me is the availability. … I don’t think there’s a week that goes by without a patient calling and saying they can’t get it anymore.’* (T11)

In this context, some GPs expressed reservations about prescriptions for patients with obesity who met the medical indication but were not dependent on the drug for glycaemic control. This became especially apparent during supply shortages, when GPs described a perceived obligation to prioritise the restricted supply:

*‘... the moral dilemma: do I prescribe a private prescription for an obesity treatment to patients while there is a supply shortage, for the regular patients, meaning diabetes patients, that was always very difficult in the beginning.’* (T02)

### Therapy adherence

GPs described patients as ‘*above average compliant*’ (T02) in the context of type 2 diabetes treatment, and attributed it to various factors. It was described as:

*‘... good because they realise, before I had a history of weight gain, and when that stops, it’s really good.’* (T07)

A further advantage was linked to the direct measurability of treatment success through HbA1c and weight levels:

*‘HbA1c is a very tangible value for patients.’* (T02)

According to the GPs, the high costs of therapy might also have influenced patients’ attitudes:

*‘And when they also know how sought after and expensive this substance is, and sometimes unavailable, I think the compliance is higher.’* (T02)

### Impact on quality of life

Some GPs also observed improvements in quality of life among patients treated with incretin mimetics in both indications, type 2 diabetes and obesity:

*‘And there are many who say: my life has completely changed because I no longer have the pressure to eat. And that addictive pressure that food can have on some people, or the addiction factor, is often just gone.’* (T10)

It was perceived that weight loss could act as a catalyst for broader lifestyle changes. GPs observed patients more motivated for physical activity and healthier habits. Psychological and psychosocial factors, notably increased self-efficacy (belief in influencing health outcomes), were considered key mechanisms behind the observed behavioural shifts:

*‘… people were motivated to exercise again. Their mood improved because they noticed that things were changing, it was working. Motivation increased.’* (T01)

In a few cases, GPs saw medication discontinued over time, with lifestyle changes maintaining weight loss:

*‘I actually have a patient who managed to lose about 15 kilos with it. And she hasn’t taken the medication for over half a year, 8 months now. And she hasn’t regained the weight.’* (T08)

GPs expressed satisfaction at offering effective treatment and seeing sustainable patient improvements:

*‘… as a doctor, you often don't get such success experiences, but with this, you can actually experience it. It feels pretty good.’* (T10)

## Discussion

### Summary

GPs perceived incretin mimetics as a new treatment option that was already gaining an established role in routine care by expanding therapeutic strategies for diabetes and obesity; while contributing to improved patient quality of life. The study design encouraged reflection on both indications, with some questions targeting diabetes or obesity specifically, and others allowing for broader comments. In practice, however, the responses were not evenly balanced. Adherence was described mainly in diabetes care, where GPs could directly compare incretin mimetics with other therapies. They perceived adherence as higher, partly due to weight loss effects. In some cases, this even initiated changes in the patients’ daily lives. Financial barriers were discussed in relation to obesity, since costs are not an issue for reimbursed diabetes prescriptions. The diabetes indication appeared more firmly embedded in clinical routines, giving GPs clearer prescribing experience, whereas in obesity this was less established and shaped mainly by patient demand or impressions. GPs reported that patients have become highly proactive in seeking these medications especially in relation to weight reduction, which was assumed to be driven by extensive media coverage. This altered the conventional doctor–patient dynamic. Challenges remained around financial accessibility, supply shortages, and concerns about misinformation or potential misuse.

### Strengths and limitations

The findings provide novel insights to the impact of incretin mimetics on the doctor–patient relationship in primary care.

The first author, a medical student in their clinical years, conducted the interviews. Her background provided sufficient medical knowledge to understand the subject matter, while her limited clinical experience reduced the risk of bias from established practice routines. Though the first author had limited experience in qualitative research, regular supervision by VvdW, NL, NK, AV, and the department’s qualitative working group ensured a professional approach.

While the sample size was limited, the participating GPs represented diverse specialties, experience, and practice characteristics. However, some potentially relevant data, such as ethnic group, were not collected.

As our findings rely on GPs’ subjective perceptions, they may be biased, for example, regarding adherence, since follow-up patients are usually more adherent. In Germany, the Disease Management Program (DMP) partly mitigates this by requiring regular check-ups for participating patients. This could support more reliable comparisons.

### Comparison with existing literature

These findings align with studies from Scandinavia. Ekenberg reported patients experienced improvements in daily life and favoured weekly injections over daily regimens, an aspect also reflected in our study.^12^ Similarly, Andreassen *et al* ^13^ observed patient demand as well as socioeconomic disparities in access, underlining relevance in different healthcare systems.

In the US, a 2025 qualitative study with primary care teams and patients described new anti-obesity medications as a transformative ‘game changer’, while also emphasising barriers to access and the need for a good patient–provider–system fit, themes also reflected in our findings. The study further highlighted patient perspectives, such as acknowledging prior weight loss efforts and distinguishing approved anti-obesity medications from drugs or supplements.^28^ As our study did not include patients, a direct comparison is not possible; however, these themes appeared to play little role for GPs.

In the UK, Keating *et al* reported positive views on integrating GLP-1 receptor agonists (GLP-1RAs), alongside concerns about patient demand and misuse. GPs felt more confident addressing weight-related issues, similar to our findings. At the same time, the UK study highlighted topics less apparent in our data, such as limited resources and fears that GLP-1RAs might be seen as a simplistic solution to complex public health challenges, diverting attention from the underlying causes of obesity.^29^

The shift from a paternalistic to a patient-centred doctor–patient relationship was pushed even further by the dynamics appearing with incretin mimetics; GPs reported patients proactively requesting them, positioning doctors more as facilitators. This shift is closely linked to shared decision making, understood as a collaborative process in which patients and clinicians jointly determine the treatment plan;^30^ and reflects broader trends in Germany, where patient-centred care is increasingly embedded in practice.^31^

Media play a key role in transforming the doctor–patient relationship by reducing information asymmetry.^32^ Research shows that health information from the internet, especially inaccurate or irrelevant content, can impede the doctor–patient relationship, reduce consultation efficiency, and increase patient confusion.^33^ Our study revealed gaps in patients’ medication knowledge, often owing to insufficient or inaccurate media information.

GPs expressed concern about misuse of these medications, which may strain the therapeutic relationship and lead to hesitancy in prescribing.

GPs reported perceived improvements in quality of life, attributed to weight loss with incretin mimetics, in both patients with diabetes — who were often additionally affected by obesity — and those with obesity alone. This perception resonates with findings from Rubino *et al,* ^34^ who demonstrated improvements in physical functioning and quality of life with semaglutide use compared with placebo. As our study captures subjective accounts rather than quantitative outcomes, we cannot establish causality, however it underscores the need for further research in this direction.

### Implications for research

Our findings highlight the need for reliable patient education and further research into real-life experiences to ensure safe and equitable use. Quantitative studies are needed to clarify adherence differences. Patient surveys could help identify factors underlying the improved adherence observed, as evidence on lived experiences, lifestyle changes, and psychosocial aspects remains limited. Studies should explore whether weight loss through these medications can act as a catalyst for behavioural change and increased self-efficacy. Future research should also compare healthcare systems across countries to investigate their influence. Finally, quantitative data are needed to capture potential benefits systematically and translate them effectively into clinical practice.

## Data

The dataset is not publicly available; however, anonymised data can be shared with other researchers on reasonable request.
